# Injuries to the Urinary Organs1Read at the fifth annual meeting of the Western New York & Pennsylvania Railway Surgeons, at Titusville, Pa., November 10, 1898.

**Published:** 1898-12

**Authors:** J. Henry Dowd

**Affiliations:** Buffalo, N. Y.; Genito-urinary surgeon to the Buffalo Hospital of the Sisters of Charity, etc., etc. 223 Franklin Street


### INJURIES TO THE URINARY ORGANS.[1]

By J. HENRY DOWD, M. D., Buffalo, N. Y.
Genito-urinary surgeon to the Buffalo Hospital of the Sisters of Charity, etc., etc.

WOUNDS of the kidneys, ureters and bladder are occasionally complications of railroad injuries and especially of the kind where compression of the abdomen and pelvis follow squeezing between buffers or freight cars.

Statistics tell us that injuries to the kidney are less common than those of the liver, all things being equal. Ehler's table of 152 cases gives subcutaneous, 90; gunshot, 50, and incised, 12. This shows that the first occurs almost twice as frequently as the open variety. The most common cause of injury is falling from a height. Other causes are blows, kicks, crushing by wheels passing over the abdomen, and falling against some stationary object. The cortex is always involved, the lesion extending in some cases to the medullary portion, causing only subcapsular hemorrhage or complete rupture of the organ. At times the lesion is as though from an incised wound, at others it is irregular or pulpy.

The peritoneum covering the kidney is rarely torn in the adult. As a general thing hemorrhage is present and it may confine itself to the substance of the kidney, the fatty covering, or break through this and extend far and wide, separating muscles and fascia, even finding its way into the peritoneal cavity.

*Symptoms.*—Of these, shock is the most pronounced. This may vary in degree from very slight to the most profound, and right here it may be well to state that shock from injuries of the kidneys and bladder is more pronounced than from wounds of any other of the internal organs. The facial expression, pulse and other indications

---

1. Read at the fifth annual meeting of the Western New York & Pennsylvania Railway Surgeons, at Titusville, Pa., November 10, 1898.

will correspond to the amount of shock present and also the hemorrhage. Pain is always present and unless masked by other injuries more severe, will be located in the affected side, possibly radiating along the course of the ureter and extending into the testicle. When the latter is very severe it is generally due to clots passing through the ureter. Hematuria is a constant symptom and by the amount it is safe to judge of the severity of the lesion. When the ureter has been ruptured the blood and urine may find its way into the abdominal cavity. Urine is diminished, and at times complete suppression takes place, but after a few days the normal quantity is reached. More or less ecchymosis is present and a prominent authority says, "the appearance of this at or near the external abdominal ring is positive evidence of kidney lesion." Infection can pass upward from the bladder and cause suppuration with symptoms accompanying such conditions.

*Treatment.*—In this, one should be prepared to combat shock, check hemorrhage or cut down and remove the injured organ at once. To fulfill these requirements rare surgical judgment is necessary. As a routine measure rest in bed, hot bottles, anodynes, sedatives and strychnine are indicated. If there is much hemorrhage no drugs are better than the free use of ergot and hamamelis. Preference should be given to a tight bandage (the injured side being padded) rather than ice packs. Vomiting must be controlled, as it facilitates bleeding.

Food should be limited in quantity and the bowels moved by enemata about every third day. It is well to put the urine in the most antiseptic condition possible, so as to avoid any possibility of infection. Guaiacol, in three to five drop doses three times a day, prevents the formation of bacteria. The most important question is, when shall we operate? Should the hemorrhage steadily increase and the patient become rapidly exsanguinated, with sighing respiration, small rapid pulse, great restlessness or large swelling rapidly form in the loin, something more heroic must be done at once.

The kidney should be cut down upon, and now only the operator can be the judge whether a rent or bleeding vessel can be secured, or if the organ should be removed. At times the bladder will fill with blood clots which will cause much discomfort, produce straining and facilitate cystitis. As it is impossible to withdraw these by the aid of a catheter, perineal section is advisable. It is positively without danger and may save the patient's life by preventing cystitis and pyelonephritis. The thermometer must be used faithfully, as this may be our first inkling of inflammatory trouble.

Injuries to the ureters generally occur at the time of operation on the uterus, but sixteen cases are reported where injury has taken place from other causes. How these small movable tubes are injured by external violence is a question yet unanswered, but it is supposed to be from the forcible pulling of the kidney or by its being pressed against the first lumbar vertebra. Early diagnosis is impossible and, except the injury be found while operating on the kidney, the only positive symptom is the appearance of a swelling, due to an accumulation of urine which leaks from the rupture. This may occur at any time from seven to thirty days after the injury. Occasionally a small amount of blood is found in the urine. There has never been an early operation reported for injured ureter, except where it occurred at the time of operation, or was found after exposing the kidney. Where such a swelling develops aspiration is necessary and should a urinary fistula result it will be necessary to cut down and close it, or, if impossible, form a new canal.

The bladder being protected as it is by the bony walls of the pelvis and in front by very elastic muscles insures its almost perfect safety from direct injury, although this at times does occur. Furthermore, the bladder when empty offers such slight resistance to external violence that it is rarely if ever injured in this condition.

Lesions may be classed under three heads: Intraperitoneal, extraperitoneal and subperitoneal. In the intraperitoneal variety it is found that the rupture usually occurs at the uppei and posterior portion, *i. e.*, where the viscus when distended comes in contact with the sacral angle. In the extraperitoneal variety the rupture is usually in front, *i. e.*, that portion exposed and partially behind the pubes. The cause of injury may well be spoken of under two conditions—namely, external and internal violence. It will only be necessary here to mention those of the second class, over-distension from whatever cause being the most prominent; straining, lifting, and the like being others.

A predisposing cause is generally present in the shape of ulceration, saculation or atony of the walls, and these secondary to some obstruction, let it be stricture or enlarged prostate.

The second or especially interesting cause to this body is some external violence applied over the bladder, such as crushing of the pelvis, kicks, sudden falls from a height, or the kneeling npon the abdomen by another. The bladder has been ruptured during childbirth, but the careful obstetrician will always see that it is empty at this time. A case has been reported of rupture during anesthesia, preparatory to operation for retention.

*Symptoms.*—The patient is liable to fall and cannot get up, complaining at the same time that he or she felt something give away inside, from which relief was experienced. Great prostration rapidly ensues, the face becomes blanched, nose pinched and pulse very rapid. Great pain is complained of over the lower abdomen, and a constant desire to urinate, with only a few drops of bloody urine escaping. A catheter if passed now will only bring forth a few drops of the same fluid. At times the extravisation of the urine is very marked, burrowing between muscles and fascia in all directions, and when intraperitoneal, is quickly followed by peritonitis. The fact should be remembered that in cases of injury to the pelvis or other parts, and again at times owing to the peculiar actions of these wounds, symptoms indicating rupture may be entirely wanting. It is, therefore, very important that a person in whom rupture or injury is suspected, be confined to bed and watched closely, for at any time the symptoms in all their fury may break forth. Outside of direct violence, such as received in a railroad or other accident, most cases of rupture of the bladder occur while the patient is intoxicated, the viscus being distended, owing to the diuretic action of alcoholic beverages. A careful history from patient or friends will aid greatly in forming a diagnosis.

*Treatment.*—Rupture of the bladder is a grave condition to deal with. Of the 315 cases collected there were but fifty-two recoveries. Of these fifty-two cases, forty-two were extraperitoneal. The question of extra- or intraperitoneal tear is often impossible of differentiation; therefore, if we can expect to give the patient any hope, the opportunity should not be lost in temporising. Localisation of the rent plays an important part. In all cases it is expedient to at once make a perineal section. We may now be positive of one thing—there will be no further extravisation of urine, and it is more than probable that we may through the perineal wound find the tear in the bladder, and thus form an opinion at once as to whether it is of intra- or extraperitoneal character. If this fails us the next step should be an incision through the abdominal wall down to the peritoneum. If urine is found we may be pretty sure that we are dealing with an extraperitoneal tear and that the contents of the bladder is escaping below the anterior fold of the membrane. It may be of great advantage if the peritoneum is gently lifted from the anterior wall of the bladder, as now urine may be found oozing up from the cellular tissue, which fills the space of Retz, this positively indicating the tear to be extraperitoneal. If no urine appears the perineum should be opened at once, the rent sought for, and when found closed tightly, the peritoneal cavity being thoroughly flushed with distilled water. It will be unnecessary to describe the technique of an operation of this description further than to say, that we should deal with these tears similar to those of the intestines, only do not let the sutures pass through the mucous membrane, so as to enter the cavity of the bladder.

In all cases a tube should be left in the bladder for at least a week, the distal end being immersed in a jar containing formaldehyde solution, which should be changed every four hours. In this way we may be able to judge as to the amount of urine passing, also as to the degree of hemorrhage.

223 Franklin Street.

